# Efficacy and safety of extracorporeal membrane oxygenation for cardiogenic shock complicating myocardial infarction: a systematic review and meta-analysis

**DOI:** 10.1186/s12872-024-03917-9

**Published:** 2024-07-16

**Authors:** Ahmed Saad Elsaeidy, Amira Mohamed Taha, Mohamed Abuelazm, Youssef Soliman, Mohamed Ahmed Ali, Abdullah K. Alassiri, Hosam Shaikhkhalil, Basel Abdelazeem

**Affiliations:** 1 Faculty of Medicine, Benha University, Benha, Egypt; 2 Faculty of Medicine, Fayoum University, Fayoum, Egypt; 3 Faculty of Medicine, Tanta University, Tanta, Egypt; 4 Faculty of Medicine, Assiut University, Assiut, Egypt; 5 Qena Faculty of Medicine, South Valley University, Qena, Egypt; 6 Faculty of Medicine, King Abdulaziz University, Jeddah, Saudi Arabia; 7 Faculty of Medicine, Islamic University of Gaza, Gaza, Palestine; 8 West Virginia University, Morgantown, WV USA

**Keywords:** ECMO, Cardiogenic shock, Myocardial infarction, Mortality, Reinfarction

## Abstract

**Background:**

Extracorporeal membrane oxygenation (ECMO) has been presented as a potential therapeutic option for patients with cardiogenic shock complicating myocardial infarction (CS-MI). We aimed to investigate the efficacy and safety of ECMO in CS-MI.

**Methods:**

A systematic review and meta-analysis synthesizing evidence from randomized controlled trials obtained from PubMed, Embase, Cochrane, Scopus, and Web of Science until September 2023. We used the random-effects model to report dichotomous outcomes using risk ratio and continuous outcomes using mean difference with a 95% confidence interval. Finally, we implemented a trial sequential analysis to evaluate the reliability of our results.

**Results:**

We included four trials with 611 patients. No significant difference was observed between ECMO and standard care groups in 30-day mortality with pooled RR of 0.96 (95% CI: 0.81–1.13, *p* = 0.60), acute kidney injury (RR: 0.65, 95% CI: 0.41–1.03, *p* = 0.07), stroke (RR: 1.16, 95% CI: 0.38–3.57, *p* = 0.80), sepsis (RR: 1.06, 95% CI: 0.77–1.47, *p* = 0.71), pneumonia (RR: 0.99, 95% CI: 0.58–1.68, *p* = 0.96), and 30-day reinfarction (RR: 0.95, 95% CI: 0.25–3.60, *p* = 0.94). However, the ECMO group had higher bleeding events (RR: 2.07, 95% CI: 1.44–2.97, *p* < 0.0001).

**Conclusion:**

ECMO did not improve clinical outcomes compared to the standard of care in patients with CS-MI but increased the bleeding risk.

**Supplementary Information:**

The online version contains supplementary material available at 10.1186/s12872-024-03917-9.

## Introduction

Cardiogenic shock (CS) is a potentially fatal condition caused by marked impairment of myocardial performance, which reduces cardiac output with subsequent end-organ hypoperfusion and hypoxia [1]. About 81% of CS cases occur following acute myocardial infarction (AMI) [2]. Despite improvements in pharmacological therapies and revascularization techniques, CS continues to be the leading cause of death in hospitalized patients with AMI [3].

Over the past years, numerous mechanical circulatory support (MCS) devices have emerged to stabilize circulation and support the heart in these patients [4]. However, strategies such as intra-aortic balloon pumping (IABP) have not proven effective, and there is little information regarding the effectiveness of other MCS devices in CS [5, 6].

Extracorporeal membrane oxygenation (ECMO) usage, a type of mechanical circulatory support, in CS management has increased by a factor of more than ten in the last decade [7]. ECMO provides full cardiopulmonary support and promptly restores organ perfusion, distinguishing it from other MCS approaches. Simpler systems and methods for nonsurgical percutaneous cannulation and vascular closure have all contributed to its increased use. ECMO patients had a reported 49% survival rate to discharge or transfer, with 58% in cases of respiratory failure and 45% in cases of cardiac failure [8]. These data, however, come from only the ELSO registered centers, leaving out information from other centers and possibly introducing a bias in the selection process.

There is limited evidence regarding the overall effect of ECMO on survival rate and its adverse effects. Numerous studies have attempted to assess the possible benefits of ECMO support, but its efficacy is still uncertain due to methodological concerns [9–11]. Further, the approximate rates of complications are still very heterogeneous, partly due to small study populations.

Current European Society of Cardiology (ESC) clinical guidelines state that MCS should be considered for hemodynamic stabilization in patients with CS as a class IIa recommendation [12]. In certain patients with refractory CS brought on by AMI, the use of ECMO is advised in a position statement from the Acute Cardiovascular Care Association of the ESC [13]. However, the majority of this advice is based on information from registry analyses and retrospective studies. Currently, there is no synthesized evidence of available data from randomized controlled trials on the use of ECMO in patients with CS.

Given the above uncertainties, this meta-analysis aims to determine the efficacy of ECMO in improving survival rates and other relevant clinical outcomes in patients with cardiogenic shock following MI. Additionally, it will evaluate the safety profile of ECMO by analyzing adverse events associated with its use.

## Methods

###  Protocol registration

This review has been registered in PROSPERO (*CRD42023486952*). The procedures for conducting this review adhered to the guidelines outlined in the Cochrane Handbook for Systematic Reviews of Interventions [14]. Furthermore, the study’s reporting followed the recommendations of the Preferred Reporting Items for Systematic Reviews and Meta-Analysis (PRISMA) statement [15].

###  Data sources & search strategy

A.S.E. and B.A. comprehensively searched MEDLINE, Embase, Cochrane Central Register of Controlled Trials (CENTRAL), SCOPUS, and Web of Science up to September 2023. They used the following keywords (Heart attack, Acute Coronary Syndrome, Myocardial Infarct*, Cardiogenic Shock, Cardiac shock, Extracorporeal Membrane Oxygenation, Extracorporeal Life Support, Extracorporeal Circulation, and Extracorporeal cardiopulmonary resuscitation). The search process involved no specific filters or limits. Additionally, we screened the reference lists of the included articles for other relevant trials. The detailed search strategy and results can be found in Table **S1**.

###  Eligibility criteria

We included the randomized controlled clinical trials (RCTs) that investigate the efficacy and safety of ECMO compared to standard care in managing CS-complicating AMI patients. Our inclusion criteria were limited to articles published in peer-reviewed international journals. We excluded observational studies, reviews, and articles that did not align with our predefined eligibility criteria.

###  Study selection

All identified studies were imported into Covidence from online databases (available via *Covidence*). The duplicates were automatically removed. Three authors (A.K.A., M.A.A., and H.S.) independently screened the title and abstract, and another author resolved the conflicts (A.S.E.). Full-text screening was performed independently by authors (A.K.A., M.A.A., and H.S.), and the conflicts were settled by another author (A.S.E). The study selection process is illustrated in a PRISMA flow chart.

###  Data extraction

Two authors (M.A.A. and H.S.) independently extracted the following data: 1- summary of the included studies (study ID, country, study design, sample size, trial procedures, ECMO weaning protocol, follow-up period); 2- baseline characteristics (sex, age, body mass index (BMI), smoking, hypertension, diabetes, hyperlipidemia, myocardial infarction, chronic heart failure, renal disease, heart rate, blood pressure, prior percutaneous coronary intervention (PCI), prior Coronary artery bypass grafting (CABG), serum lactate, serum creatinine, infarct-related artery) 3- outcomes data (30-day mortality, 30-day reinfarct, bleeding, stroke, sepsis, pneumonia, and acute kidney injury). Any conflict was handled through discussion or by inviting A.S.E. to make a final decision.

###  Risk of bias and certainty of evidence

Using ROB-II, (A.K.A. and H.S.) assessed the quality of the included studies [16]. ROB-II investigates the risk of bias according to five domains: 1- Randomization process; 2- Deviations from intended interventions; 3- Missing outcome data; 4- Outcome measurement; 5- Selection of the reported result. Any conflict was handled through discussion or by inviting A.S.E. to make a final decision.

Furthermore, (M.A.) applied the Grading of Recommendations Assessment, Development, and Evaluation (GRADE) guidelines to appraise the quality of evidence [17, 18]. GRADE framework appraises the quality of evidence according to various factors, including imprecision, indirectness, inconsistency, publication bias, and risk of bias. This evaluation was carried out for each outcome, and the decisions made were appropriately justified and documented.

###  Statistical analysis

The meta-analysis was performed using the Review Manager (RevMan) software. We calculated the risk ratio (RR) along with its corresponding 95% Confidence interval (CI) to evaluate diverse outcomes. The fixed-effects model was employed. Heterogeneity was assessed using the I^2^ and Chi-square tests; the Chi-square test determined substantial heterogeneity with an alpha level below 0.1, following the Cochrane Handbook for Systematic Reviews and Interventions [14], while the I^2^ test interpretation is as follows: 0–40% (not significant), 30–60% (moderate heterogeneity), and 50–90% (considerable heterogeneity). The overall effect size was deemed statistically significant if the *P*-value was < 0.05.

Moreover, we implemented a trial sequential analysis (TSA) in light of the relatively small number of studies included in our study and to evaluate the reliability of our results. The TSA approach was employed to balance type I and type II errors, providing an estimate of when the effect size would be substantial enough to withstand the impact of additional studies. Consequently, TSA enhances transparency and informed decision-making in our meta-analysis. This method helps clarify the level of certainty in our findings and whether further studies are needed for confirmation [19, 20].

##  Results

###  Search results and study selection

**Fig. 1:**
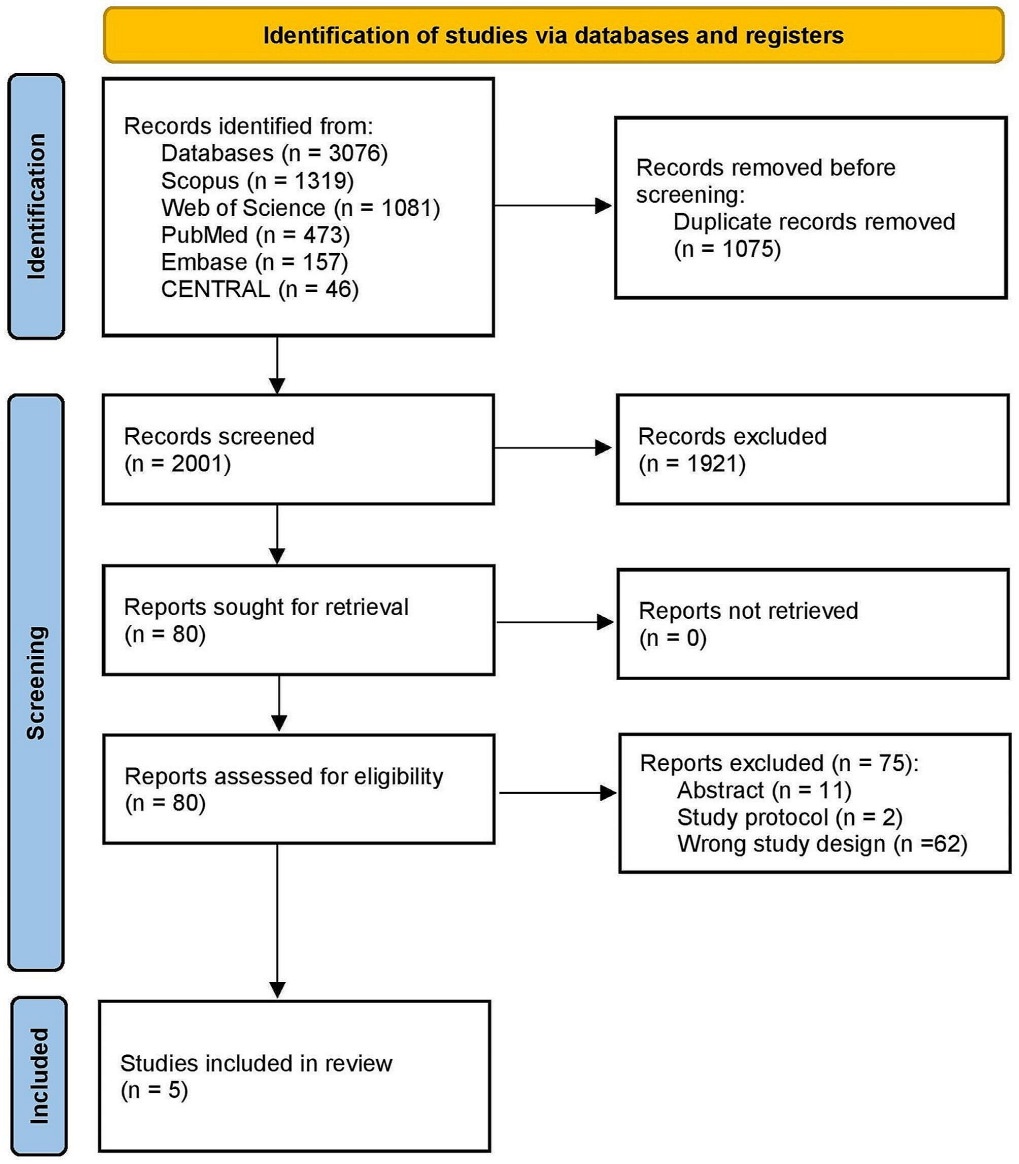
PRISMA flow chart of the screening process

A total of 3.076 records were initially identified after comprehensive searches on databases. After eliminating duplicate entries, we were left with 2.001 studies eligible for title and abstract screening. Among these, 1.921 studies were excluded due to their lack of relevance to our research objectives. Consequently, 80 articles proceeded to full-text screening. During this phase, 75 were excluded based on specific criteria, including four RCTs published in five publications [21–25] in our systematic review and meta-analysis. The PRISMA flow diagram in the study is illustrated in Fig. 1.

###  Characteristics of included studies

A total of four open-label RCTs that involved a total of 611 patients were included [21–24]. Three studies were multicenter, while only Lackermair et al. 2020 was a single-center RCT. All studies’ follow-up period was 30 days [21–24]. Two studies involved additional one-year follow-up [23, 24]. Cardiogenic shock was defined as a systolic blood pressure of < 90 mmHg for > 30 min (or if the catecholamines were needed to maintain the systolic blood pressure > 90 mmHg) and > 3 mmol/L arterial lactate level with signs of organ hypoperfusion with at least one of the following: 1- altered mental status; 2- cold or clammy skin and limbs; 3- urine output of < 30 ml/h [19–22].

A summary of the included studies with more details about the interventions and ECMO weaning protocol is shown in Table 1. Most patients were male (80.19%), with a mean age over 60 years. We found that 57.6% of the patients had a prior history of hypertension. 13% of patients had previous PCI, whereas 2.65% had previous CABG. The baseline characteristics of included patients with more details about the medical history and clinical parameters are shown in Table 2.

**Table 1:** Summary of the included studies

Study ID	Country	Study design	Sample size	Trial procedure		ECMO Weaning Protocol	Follow-up Period
				ECMO group	Standard-Care group		
Thiele et al. 2023	Germany, Slovenia	Phase 3, Open-label RCT	417	ECLS began during index catheterization, preferably before PCI. An antegrade arterial femoral sheath was advised to reduce the risk of lower limb ischemia.	The study protocol forbade control group to ECLS crossover. An intraaortic balloon pump or microaxial transvalvular flow pump can be used for escalation therapy under certain hemodynamic circumstances. These criteria included severe hemodynamic instability with impending collapse, a 6-hour arterial lactate elevation of more than 3 mmol per liter, or a 50% increase in vasopressor use from baseline to maintain a mean arterial blood pressure over 65 mm Hg.	I. Hemodynamic stability means sustained systolic blood pressure > 90 mmHg or mean blood pressure > 65 mmHg (> 60 min). II. Persistent ejection with little inotropic support III. No severe hypoxemia IV. Arterial lactate < 2 mmol/l V. Central venous saturation > 65% ECLS flow should be reduced by 0.5-1.0 l/min over 4–6 h if the conditions are met. Successful weaning with subsequent ECLS removal was defined as flow reduction to 1.0-1.5 l/min. Removal was to be performed according to local expertise. Percutaneous closure was recommended	30 days
Ostadal et al. 2022 (ECMO-CS)	Czech Republic	Phase 3, Open-label RCT	117	Except for immediate VA-ECMO implementation in the intervention group, all other diagnostic and therapeutic procedures were performed according to current standards, including percutaneous coronary or noncoronary intervention, cardiac surgery, and mechanical circulatory support.	If serum lactate rises by 3 mmol/L from the lowest value in the last 24 h, VA-ECMO may be utilized downstream in the early conservative group. The protocol did not specify left ventricular venting during VA-ECMO support or limb ischemia prevention or treatment and were left to physicians.	NA	30 days
Banning et al. 2023 (EURO SHOCK)	Spain, Germany, United Kingdom, Norway, Latvia, Belgium	Open-label RCT	35	Intra-aortic balloon pump (IABP) use was permitted as a left ventricular unloading in patients receiving VA-ECMO therapy.	IABP was allowed in the control therapy group, but mechanical support devices were discouraged. However, it was permitted if the physicians felt it would help in clinically deteriorated cases and acknowledged as procedural violation.	Removal was to be performed according to local expertise and the patient’s clinical status by intensive care physicians.	30-days and 1-year.
Lackermair et al. 2020 (ECLS-Shock-I)	Germany	phase 4, Open-label RCT	42	ECLS implantation was performed in the catheterization laboratory under fluoroscopic control. To prevent lower limb ischemia, a distal limb perfusion cannula was inserted into the superficial femoral artery. Stöckert Centrifugal Pump System (SCP, LivaNova, Munich, Germany) used for mechanical circulation support.	NA	Weaning was done after sustained hemodynamic stabilization on low levels of inotropic and vasopressor support with sufficient peripheral perfusion and end-organ function had been achieved.	30-days and 1-year.

ECLS: Extra-Corporeal Life Support; IABP: Intra-aortic balloon pump; NA: Not Avaible data; RCT: Randomized controlled trial; VA-ECMO: Venoarterial-Extracorporeal membrane oxygenation;

###  Risk of bias and certainty of evidence

**Fig. 2:**
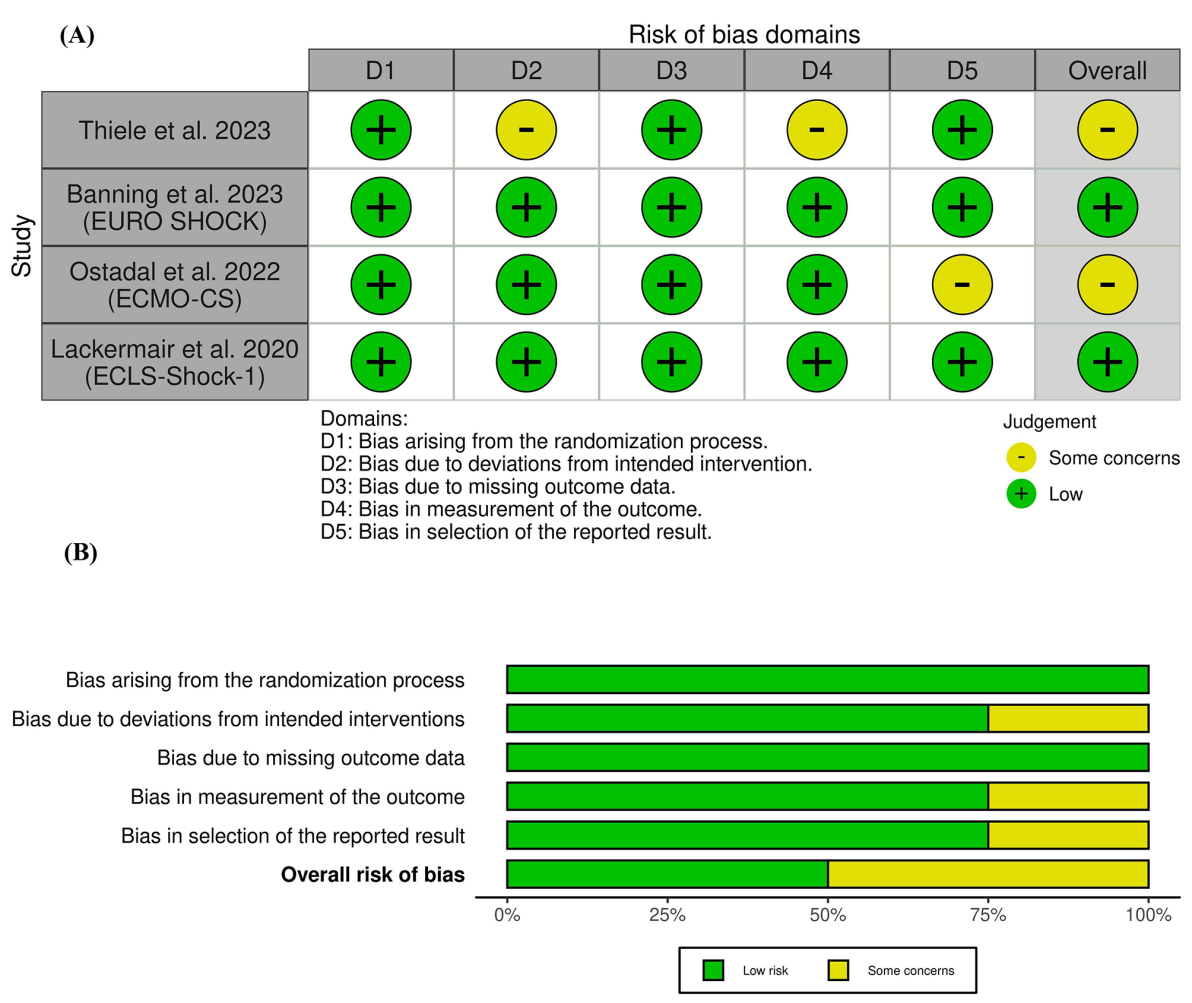
Quality assessment of risk of bias in the included trials. The upper panel (**A**) presents a schematic representation of risks (low = green, unclear = yellow, and high = red) for specific types of biases of each study in the review. The lower panel (**B**) presents risks (low = green, unclear = yellow, and high = red) for the subtypes of biases of the combination of studies included in this review

According to the ROB-2 tool, two studies demonstrated an overall low risk of bias, and two had an overall some concerns of bias [21–24], as shown in Fig. 2. We had some concerns about deviations from the intended intervention and outcome measurement in Thiele et al. 2023 [21] and about the selection of the reported results in Ostadal et al. 2023 [22]. We found a low risk of bias arising from the randomization process or missing outcome data, but there were some concerns about bias due to deviations from the intended intervention, outcome measurement, and selection of the reported results. The authors’ notes about each item in ROB-II are further clarified in **Table S2**.

**Table 2:** Baseline characteristics of the included studies

Study ID		General characteristics			Medical history							
		Sex (Male), N (%)	Age (Years), Mean ± SD	BMI, Mean ± SD	Smoking (Current), N (%)	Hypertension, N (%)	Diabetes, N (%)	Hyperlipidemia, N (%)	Myocardial infarction, N (%)		Chronic heart failure, N (%)	Renal disease, N (%)
									STEMI	NSTEMI		
Thiele et al. 2023	ECMO	170 (81.3)	62.3 ± 9.7	27.3 ± 3.7	74 (36.3)	118 (57)	70 (33.7)	55 (26.6)	23 (11.1)		NA	NA
	Standard Care	169 (81.2)	63.7 ± 10.5	28 ± 4.5	71 (34.5)	115 (55.8)	60 (29.1)	74 (35.9)	31 (15.0)		NA	NA
Ostadal et al. 2022 (ECMO-CS)	ECMO	43 (74.1)	67 ± 10.6	NA	14 (25.9)	35 (62.5)	16 (28.6)	NA	30 (51.7)	7 (12.1)	14 (25.0)	7 (12.5)
	Standard Care	43 (72.9)	64.7 ± 9.9	NA	27 (47.4)	38 (65.5)	21 (36.2)	NA	29 (49.2)	7 (11.9)	13 (22.4)	9 (15.8)
Banning et al. 2023 (EURO SHOCK)	ECMO	13 (81.3)	66 ± 9	27 ± 5	4 (28.6)	10 (66.7)	7 (50)	6 (66.7)	0 (0)	1 (6.7)	0 (0)	1 (7.7)
	Standard Care	16 (88.9)	65 ± 12	28 ± 4	6 (35.3)	10 (71.4)	5 (31.3)	7 (87.5)	1 (6.7)	2 (13.3)	0 (0)	4 [25]
Lackermair et al. 2020 (ECLS-Shock-I)	ECMO	16 (76.2)	60 ± 14.3	26.6 ± 2.5	9 (45)	11 (55)	3 [15]	5 [25]	13 (61.9)	8 (38.1)	NA	NA
	Standard Care	20 (95)	68 ± 11.1	27.3 ± 4.8	11 (55)	15 (75)	6 [30]	10 (50)	8 (38.1)	13 (61.9)	NA	NA
Study ID		Clinical Parameters										
		Heart rate (beat/min), Mean ± SD	Blood pressure (mm Hg), Mean ± SD		Prior PCI, N (%)	Prior CABG, N (%)	Serum lactate (mmol/L), Mean ± SD	Serum creatinine (mg/dl), Mean ± SD	Infarct-related artery, N (%)			
			Systolic	Diastolic					Left coronary artery	Left anterior descending	Left circumflex	Right coronary artery
Thiele et al. 2023	ECMO	91.7 ± 26	98.3 ± 29.9	61.3 ± 17.2	27 (13.0)	5 (2.4)	7 ± 3.8	1.2 ± 0.4	20 (9.9)	95 (46.8)	36 (17.7)	52 (25.6)
	Standard Care	92 ± 29	99 ± 29.9	60.3 ± 15.7	43 (20.9)	6 (2.9)	7.2 ± 4	1.2 ± 0.37	20 (10.0)	97 (48.5)	35 (17.5)	48 (24.0)
Ostadal et al. 2022 (ECMO-CS)	ECMO	110 ± 32.22	86.3 ± 11.4	NA	NA	NA	5.6 ± 4	NA	NA	NA	NA	NA
	Standard Care	100 ± 20.7	91.2 ± 19.4	NA	NA	NA	5.1 ± 3.1	NA	NA	NA	NA	NA
Banning et al. 2023 (EURO SHOCK)	ECMO	N/A	90 ± 23	55 ± 15	2 (13.3)	0 (0)	5.9 ± 3.7	1.2 ± 0.4	2 (12.5)	7 (43.75)	2 (12.5)	5 (31.3)
	Standard Care	N/A	107 ± 38	68 ± 27	5 (31.3)	1 (6.3)	8.2 ± 4.6	1.5 ± 0.5	4 (22.2)	8 (44.4)	1 (5.6)	4 (22.2)
Lackermair et al. 2020 (ECLS-Shock-I)	ECMO	83.3 ± 23.9	116.7 ± 23.9	71.3 ± 12.7	4 [20]	0 (0)	5.3 ± 4.6	1.3 ± 0.2	1 (5%)	15 (72%)	2 (10%)	3 (14%)
	Standard Care	80.7 ± 22.3	102.7 ± 19.1	66 ± 18.3	4 [20]	0 (0)	6 ± 4.7	1.3 ± 0.2	3 (14%)	8 (38%)	5 (24%)	5 (24%)

Data is presented as Mean ± Standard deviation or as Freqancy (Precentage)

BMI: Body mass index; CABG: Coronary artery bypass graft; ECMO: Extracorporeal membrane oxygenation; N: Number; NA: Not Avaible data; NSTEMI: Non-ST-elevation myocardial infarction; PCI: Percutaneous coronary intervention; STEMI: ST-elevation myocardial infarction;

The GRADE system declared that the 30-day mortality rate and bleeding events yielded moderate-quality evidence. The other outcomes yielded low-quality evidence, except pneumonia, which yielded very low-quality evidence. Details and explanations are clarified in Table 3.

**Table 3:** GRADE evidence profile

Certainty assessment							Summary of findings				
Participants (studies) Follow-up	Risk of bias	Inconsistency	Indirectness	Imprecision	Publication bias	Overall certainty of evidence	Study event rates (%)		Relative effect (95% CI)	Anticipated absolute effects	
							With Standard of Care	With VA-ECMO		Risk with Standard of Care	Risk difference with VA-ECMO
**30-day mortality**											
611 (4 RCTs)	not serious	not serious	not serious	serious^a^	none	⨁⨁⨁◯ Moderate	148/306 (48.4%)	140/305 (45.9%)	**RR 0.96** (0.81 to 1.13)	484 per 1,000	**19 fewer per 1,000** (from 92 fewer to 63 more)
**30-day reinfarct**											
491 (3 RCTs)	not serious	not serious	not serious	very serious^b^	none	⨁⨁◯◯ Low	5/247 (2.0%)	4/244 (1.6%)	**RR 0.95** (0.25 to 3.60)	20 per 1,000	**1 fewer per 1,000** (from 15 fewer to 53 more)
**Stroke**											
608 (4 RCTs)	not serious	not serious	not serious	very serious^b^	none	⨁⨁◯◯ Low	11/306 (3.6%)	12/302 (4.0%)	**RR 1.16** (0.38 to 3.57)	36 per 1,000	**6 more per 1,000** (from 22 fewer to 92 more)
**Acute kidney injury/Renal Replacement Therapy**											
566 (3 RCTs)	not serious	not serious	not serious	very serious^b^	none	⨁⨁◯◯ Low	40/285 (14.0%)	25/281 (8.9%)	**RR 0.65** (0.41 to 1.03)	140 per 1,000	**49 fewer per 1,000** (from 83 fewer to 4 more)
**Bleeding events**											
608 (4 RCTs)	not serious	not serious	not serious	serious^a^	none	⨁⨁⨁◯ Moderate	36/306 (11.8%)	76/302 (25.2%)	**RR 2.07** (1.44 to 2.97)	118 per 1,000	**126 more per 1,000** (from 52 more to 232 more)
**Sepsis**											
611 (4 RCTs)	not serious	not serious	not serious	very serious^b^	none	⨁⨁◯◯ Low	51/306 (16.7%)	54/305 (17.7%)	**RR 1.06** (0.77 to 1.47)	167 per 1,000	**10 more per 1,000** (from 38 fewer to 78 more)
**Pneumonia**											
152 (2 RCTs)	not serious	not serious	not serious	extremely serious^b^	none	⨁◯◯◯ Very low	19/77 (24.7%)	18/75 (24.0%)	**RR 0.99** (0.58 to 1.68)	247 per 1,000	**2 fewer per 1,000** (from 104 fewer to 168 more)

CI: confidence interval; RR: risk ratio

Explanations

a. Low number of events < 300 event

b. A wide confidence interval that does not exclude the risk of appreciable harm or benefit and a low number of events < 300 events

###  Efficacy outcomes

####  30-day mortality

**Fig. 3:**
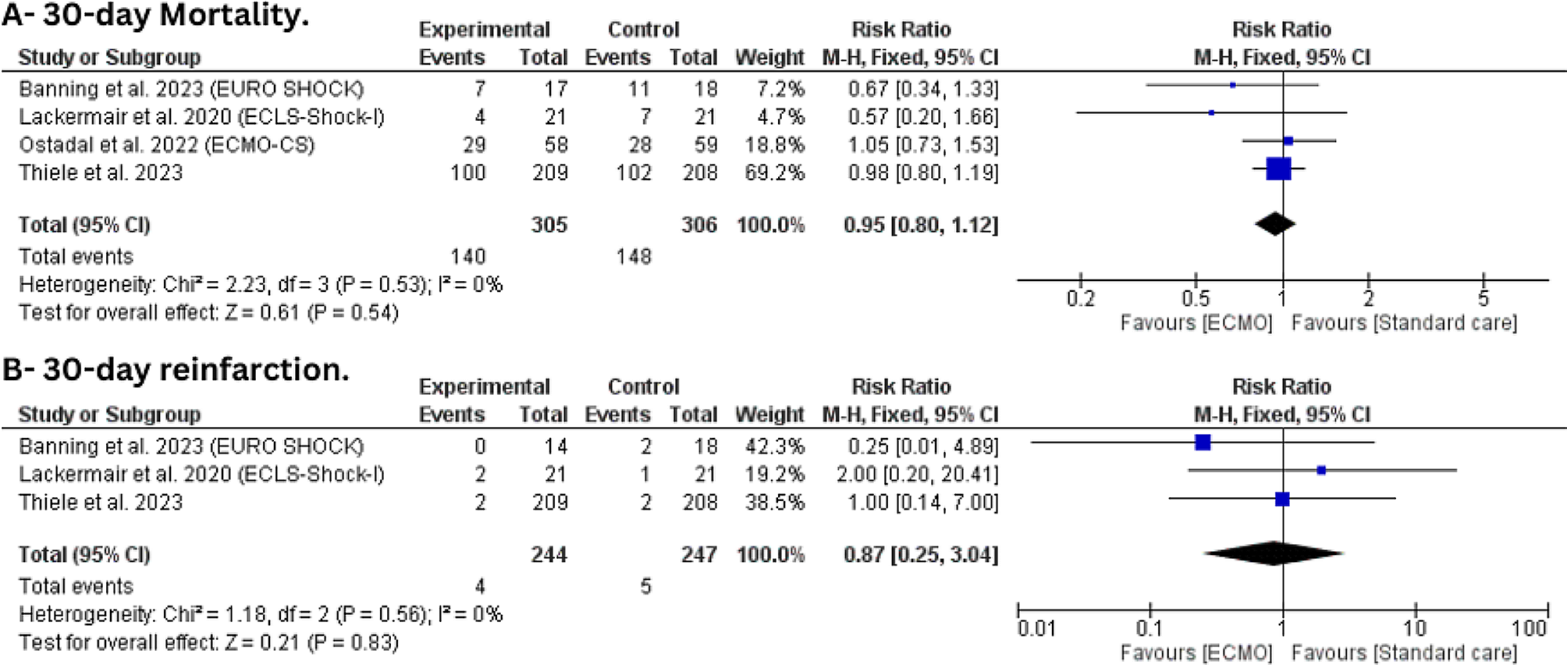
Forest plot of efficacy outcomes (**A**) 30-day mortality and (**B**) 30-day reinfarction

The Four trials with 611 patients were included in this outcome [21–24]. The 30-day mortality rate in the ECMO group was 45.9%, while 48.4% in the standard care group. However, There was no statistically significant difference between ECMO and standard care (RR: 0.95, 95%CI [0.80, 1.12], *P* = 0.54) with no statistically significant heterogeneity (I^2^ = 0%, *P* = 0.53) (Fig. 3-A).

#### 30-days reinfarction

Three trials with 491 patients were included in this outcome [21, 23, 24]. The trials state that only four patients in the ECMO group and five patients in the standard care group had re-infarcted in 30 days. We found no statistically significant difference between ECMO and conservative treatment (RR: 0.87, 95%CI [0.25, 3.04], *P* = 0.83) with no statistically significant heterogeneity (I^2^ = 0%, *P* = 0.56) (Fig. 3-B).

### Safety outcomes

#### Stroke

**Fig. 4:**
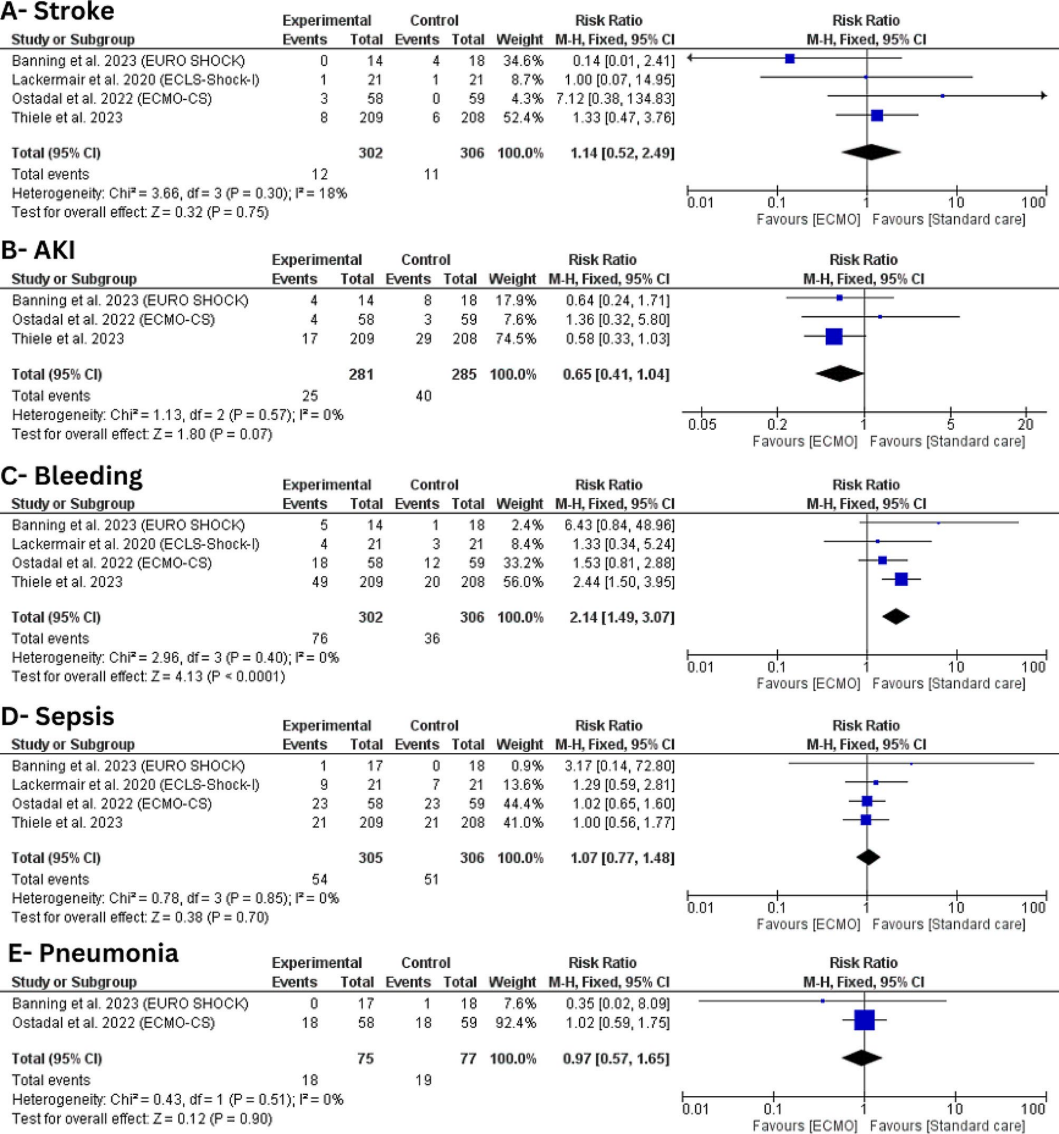
Forest plot of safety outcomes (**A**) stroke; (**B**) acute kidney injury (AKI); (**C**) bleeding; (**D**) sepsis; (**E**) pneumonia

Four trials with 608 patients were included in this outcome [21–24]. There was no statistically significant difference between ECMO and conservative treatment (RR: 1.14, 95%CI [0.52, 2.49], *P* = 0.75) with no statistically significant heterogeneity (I^2^ = 18%, *P* = 0.30) (Fig. 4-A).

#### Acute kidney injury

Three trials with 566 patients were included in this outcome [21, 22, 24]. There was no statistically significant difference between ECMO and conservative treatment (RR: 0.65, 95%CI [0.41, 1.04], *P* = 0.07) with no statistically significant heterogeneity (I^2^ = 0%, *P* = 0.57) (Fig. 4-B).

####  Bleeding

**Fig. 5:**
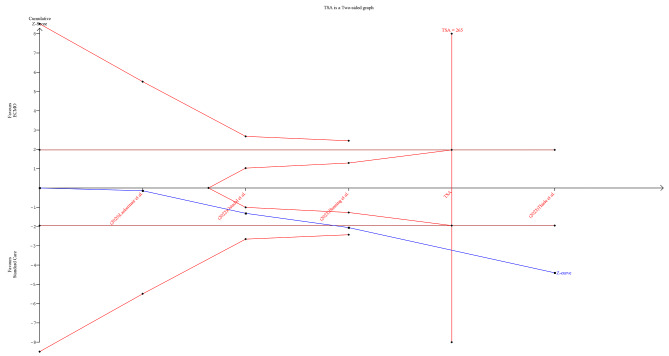
Trial sequential analysis of bleeding outcome

On the other hand, ECMO was found to be associated with a statistically significant higher risk of bleeding (RR: 2.14, 95%CI [1.49, 3.07], *P* < 0.0001) compared to conservative care with no statistically significant heterogeneity (I^2^ = 0%, *P* = 0.40) (Fig. 4-C). TSA was performed, and it revealed that the Z-curve had crossed the conventional boundary and entered the area of harm, indicating that ECMO has shown a statistically significant impact on increased bleeding risk, prompting potential concerns necessitating further evaluation (Fig. 5).

####  Sepsis

Four trials with 611 patients were included in this outcome [21–24]. There was no statistically significant difference between ECMO and conservative treatment (RR: 1.07, 95%CI [0.77, 1.48], *P* = 0.85) with no statistically significant heterogeneity (I^2^ = 0%, = 0.85) (Fig. 4-D).

####  Pneumonia

Only two trials with 152 patients were included in this outcome [22, 24]. We found no statistically significant difference between ECMO and conservative treatment (RR: 0.97, 95%CI [0.57, 1.65], *P* = 0.90) with no significant heterogeneity (I^2^ = 0%, *P* = 0.51) (Fig. 4-E).

##  Discussion

This meta-analysis comprehensively assesses the efficacy and safety of ECMO for CS-complicating MI. The pooled analysis of four randomized controlled trials found that ECMO has not substantially decreased 30-day mortality or reduced the risk of 30-day reinfarction in patients with CS after MI. Regarding safety outcomes, using ECMO significantly increases the risk of bleeding. At the same time, there was no significant rise in the risk of pneumonia, sepsis, stroke, or acute kidney injury with ECMO.

Despite the increasing use of ECMO, its benefits and risks remain undetermined, particularly when compared with other conservative treatment methods. In the recent ECLS-SHOCK trial, Thiele and colleagues aimed to determine if early routine ECLS therapy in patients experiencing acute MI complicated by CS and undergoing early planned revascularization enhances survival compared to standard medical therapy [21]. Out of 420 randomized patients, 417 were analyzed, revealing a similar 30-day mortality rate between the ECLS group (47.8%), which is close to the 30-day mortality rate in our analysis (45.9%), and the control group (49.0%) with insignificant difference (*P* = 0.81). Further, the incidence of myocardial reinfarction was almost similar between both arms (2/209 in the ECMO group vs. 2/208 in the control group), which aligns with our findings.

EURO SHOCK trial, a multicentric study that included 35 patients with cardiogenic shock, revealed that the incidence of mortality from all causes at thirty days was 43.8% in the group receiving ECMO as opposed to 61.1% in the group receiving standard treatment (hazard ratio [HR] of 0.56 with a 95%CI ranging from 0.21 to 1.45; *p* = 0.22) [24]. After a year, the all-cause mortality rate for patients receiving ECMO was 51.8%, whereas 81.5% for patients receiving standard therapy (HR 0.52, 95%CI: 0.21–1.26; *p* = 0.14). These findings are consistent with ours and may imply that, while ECMO might stabilize hemodynamics, its effects do not always translate into improved short-term survival or reduced recurrence of myocardial infarction. This could be due to the extent of the underlying cardiac injury as well as the challenges associated with CS management.

In a previous meta-analysis, a propensity-matched analysis of 438 patients found that ECLS use resulted in a 13% increase in 30-day survival and a 14% improvement in favorable neurological outcomes in CS cases [26]. When compared to IABP, ECLS resulted in a 33% higher 30-day survival rate in patients with cardiogenic shock following a myocardial infarction. Still, there was no significant difference when compared to TandemHeart/Impella. The study revealed that ECLS significantly enhances the likelihood of obtaining a positive neurological outcome, as measured by a Cerebral Performance Category (CPC) score of 1 or 2 [24]. Improvement was observed at the 30-day mark and during a more prolonged follow-up period (14% and 11% risk difference, respectively) [24].

The findings indicate that ECLS efficacy in cases of CS varies depending on the comparator treatment. The contrasting results between Ouweneel et al. analysis and our analysis could be attributed to different study designs, as we included RCTs only, while the previous meta-analysis focused on observational studies.

A more recent meta-analysis incorporated thirty-two studies encompassing a total of 12,756 patients with CS [27]. The authors revealed that a significant portion of patients, specifically 62% (8,493 out of 12,756), encountered in-hospital mortality. Notably, over one-third of these patients died while receiving ECMO support. Univariate meta-regression analyses identified in-hospital mortality as being associated with factors such as patient age exceeding 60 years, shorter ECMO support duration, and the presence of infection.

When compared to conservative care, ECMO was associated with a statistically significant increased risk of bleeding, which raises important clinical implications. Bleeding complications can be fatal and necessitate immediate medical attention. Clinicians must carefully balance the benefits of ECMO with the elevated bleeding risk, especially in patients who are at high risk of bleeding. In addition, our trial sequential analysis suggests that ECMO could significantly impact bleeding risk, emphasizing the importance of close monitoring and timely intervention to reduce this risk.

Rajsic et al. reported bleeding as the second most frequent adverse event, 49% (1,971 out of 4,523) after renal failure, affecting 51% (693 out of 1,351) [27]. Further, this bleeding incidence was similar to that reported by Zangrillo et al., who reported any bleeding event in 40% of patients [28]. This high bleeding rate in the ECMO group could be multifactorial, resulting from various factors related to the treatment, patient characteristics, and the underlying clinical issue. Prolonged anticoagulation is necessary to prevent the formation of clots within the circuit, which is essential for maintaining the ECMO flow of blood and preventing clot-related adverse effects. Prolonged use of anticoagulation and the mechanical strain exerted on the circulatory system during ECMO support may exacerbate bleeding tendencies. Extended ECMO support periods include placing large cannulas into major blood vessels, which carry inherent bleeding hazards during insertion and subsequent usage. Minimizing complications related to bleeding and maintaining anticoagulation balance require efficient clinical management.

Our study found no statistically significant difference in the probability of stroke between patients who received ECMO and those who were managed conservatively. This finding is reassuring, as there have been concerns imposed about the potential for ECMO to raise the risk of stroke [29]. However, this result must be interpreted in light of the particular patient’s condition and clinical indications for ECMO. While the average risk of stroke is not increased, specific patient characteristics and underlying diseases could have a substantial part in stroke risk.

Xie et al. pooled 22 observational studies for patients who received ECMO for refractory CS or CA [30]. Survival rates at 3, 6, and 12 months were 55.9%, 47.6%, and 54.4%, respectively. Complication rates were particularly substantial for renal impairment (47.4%), infection (25.1%), and neurologic deficits (13.3%). We found a statistically insignificant difference in AKI risk between the ECMO and conservative treatment groups, suggesting that ECMO does not increase the risk of AKI intrinsically. However, it is critical to acknowledge that a variety of variables, such as hemodynamic instability, the use of nephrotoxic drugs, and the extent of the underlying disease, can cause AKI. Regarding these outcomes, future large RCTs are required to assist these findings.

###  Strengths and limitations

We established clear inclusion criteria specifying the study design (RCTs), patient population (those with MI complicated with CS), interventions (ECMO or ECLS), and comparators (standard care). The absence of significant heterogeneity between the studies included in these outcomes suggests that the findings are consistent concerning various study populations and settings. However, the relatively small number of included studies (four trials) in the meta-analysis might impact the generalizability of the findings and the ability to detect significant differences in some outcomes. Hence, we applied TSA to assess data reliability and certainty, particularly in light of a limited number of studies, enhancing transparency and informed decision-making in our meta-analysis. Also, the GRADE framework considerably improves the use of meta-analysis in healthcare research by assessing the quality of evidence and the strength of recommendations and analyzing many aspects of evidence quality, such as bias and consistency. By applying the GRADE framework, we aim to provide a more informed basis for clinical practice and policy formulation. In summary, while we acknowledge the overlap with previous studies and the limitation of the number of included trials, our use of TSA and GRADE methodology enhances the rigor and reliability of our meta-analysis, contributing valuable insights to the understanding of outcomes in cardiogenic shock patients placed on ECMO devices.

Furthermore, our analysis extends beyond the 30-day outcomes examined in Zeymer et al. [31], providing valuable insights into the safety of ECMO support over a longer duration, up to one year. By focusing on this extended timeframe, we offer clinicians and researchers a comprehensive understanding of the sustained safety profile of ECMO therapy in cardiogenic shock patients.

While the outcomes discussed in the Zeymer et al. [31] paper are undoubtedly valuable, our study complements them by providing a more detailed exploration of the longer-term consequences and safety considerations associated with ECMO intervention. Thus, our research contributes to the existing literature by filling the gap in knowledge regarding the extended safety outcomes of ECMO therapy in this patient population. However, the predominance of the ELCS-SHOCK trial data in our analysis might affect the reliability of our findings.

###  Conclusion

While ECMO can be a valuable tool for hemodynamic support, our findings suggest that the current recommendation for ECMO use in CS post-MI should be reconsidered. The lack of a clear survival benefit and significant bleeding risks calls for a more cautious approach to ECMO use. Future RCTs are needed to understand better patient subgroups that could benefit most from ECMO and possible approaches to mitigate its risks.

### Electronic supplementary material

Below is the link to the electronic supplementary material.

Supplementary Material 1

## Data Availability

The datasets used and/or analyzed analysis during the current study are available from the corresponding author upon reasonable request.
