# Supplementary material for: Efficacy and safety of extracorporeal membrane oxygenation for cardiogenic shock complicating myocardial infarction: a systematic review and meta-analysis
Source: BMC Cardiovasc Disord. 2024 Jul 16;24:362. doi: 10.1186/s12872-024-03917-9 (PMC11251331; doi:10.1186/s12872-024-03917-9)
Supplement: Supplementary file 1 — Supplementary Material 1 [file 12872_2024_3917_MOESM1_ESM.docx]

**Efficacy and safety of extracorporeal membrane oxygenation for cardiogenic shock complicating myocardial infarction: A systematic review and meta-analysis.**

**Running title:** ECMO for MI-Cardiogenic Shock.

Ahmed Saad Elsaeidy ^1*^, Amira Mohamed Taha ^2^ (Co-First author), Mohamed Abuelazm ^3^, Youssef Soliman ^4^, Mohamed Ahmed Ali ^5^, Abdullah K. Alassiri ^6^, Hosam Shaikhkhalil ^7^, Basel Abdelazeem ^8^

1- Faculty of Medicine, Benha University, Benha, Egypt.

[Ahmed.Saad.Elsaeidy@gmail.com](mailto:Ahmed.Saad.Elsaeidy@gmail.com)

<https://orcid.org/0000-0002-1643-9750>

2- Faculty of Medicine, Fayoum University, Fayoum, Egypt.

[am7529@fayoum.edu.eg](mailto:am7529@fayoum.edu.eg)

<https://orcid.org/0000-0002-9620-7350>

3- Faculty of Medicine, Tanta University, Tanta, Egypt.

[dr.mabuelazm@gmail.com](mailto:dr.mabuelazm@gmail.com)

<https://orcid.org/0000-0002-2514-0689>

4- Faculty of Medicine, Assiut University, Assiut, Egypt.

[youssefrefaat138@gmail.com](mailto:youssefrefaat138@gmail.com)

<https://orcid.org/0000-0003-4204-3706>

5- Qena Faculty of Medicine, South Valley University, Qena, Egypt.

[mohammedahmedalihassan2003@gmail.com](mailto:mohammedahmedalihassan2003@gmail.com)

<https://orcid.org/0000-0001-8142-8073>

6- Faculty of Medicine, King Abdulaziz University, Jeddah, Saudi Arabia.

[Assiri1617@gmail.com](mailto:Assiri1617@gmail.com)

<https://orcid.org/my-orcid?orcid=0000-0002-3799-7313>

7- Faculty of Medicine, Islamic University of Gaza, Gaza, Palestine.

[hosam.shaikhkhalil@gmail.com](mailto:hosam.shaikhkhalil@gmail.com)

<https://orcid.org/0000-0003-1384-3886>

8- West Virginia University, Morgantown, West Virginia, USA.

[Baselelramly@gmail.com](mailto:Baselelramly@gmail.com)

<https://orcid.org/0000-0002-2919-6196>

***- Corresponding author.**

Ahmed Saad Elsaeidy

Faculty of Medicine, Benha University, Benha, Egypt.

Ahmed.Saad.Elsaeidy@gmail.com

<https://orcid.org/0000-0002-1643-9750>

**Table of Contents**

[Table S1. Details of searching Databases. 3](#_Toc162275123)

[Table S2. Details of Quality Assessment Using ROB-II Tools. 4](#_Toc162275124)

| Table S1. Details of searching Databases. | | |
| --- | --- | --- |
| **Database & search date** | **Results** | **Search strategy** |
| Pubmed  13 Sep 2023 | 473 | (((((Myocardial Infarct*[Title/Abstract]) OR (Heart Attack[Title/Abstract])) OR (Acute Coronary Syndrome[Title/Abstract])) OR (Coronary occlusion[Title/Abstract])) AND ((Cardiogenic Shock[Title/Abstract]) OR (Cardiac shock[Title/Abstract]))) AND ((((Extracorporeal Membrane Oxygenation[Title/Abstract]) OR (Extracorporeal Life Support[Title/Abstract])) OR (Extracorporeal Circulation[Title/Abstract])) OR (Extracorporeal cardiopulmonary resuscitation[Title/Abstract])) |
| Web Of Science  13 Sep 2023 | 1081 | #1  TS=((((Myocardial Infarct*) OR (Heart Attack)) OR (Acute Coronary Syndrome)) OR (Coronary occlusion))  #2  TS=((Cardiogenic Shock) OR (Cardiac shock))  #3  TS=((((Extracorporeal Membrane Oxygenation) OR (Extracorporeal Life Support)) OR (Extracorporeal Circulation)) OR (Extracorporeal cardiopulmonary resuscitation))  #4  #1 AND #2 AND #3 |
| Scopus  13 Sep 2023 | 1,319 | ( TITLE-ABS-KEY ( ( ( ( myocardial infarct* ) OR ( heart attack ) ) OR ( acute coronary syndrome ) ) OR ( coronary occlusion ) ) ) AND ( TITLE-ABS-KEY ( ( cardiogenic shock ) OR ( cardiac shock ) ) ) AND ( TITLE-ABS-KEY ( ( ( ( extracorporeal membrane oxygenation ) OR ( extracorporeal life support ) ) OR ( extracorporeal circulation ) ) OR (extracorporeal cardiopulmonary resuscitation))) |
| Cochrane library  13 Sep 2023 | 46 | (((Extracorporeal Membrane Oxygenation) OR (Extracorporeal Life Support)) OR (Extracorporeal Circulation)) OR (Extracorporeal cardiopulmonary resuscitation) in Title Abstract Keyword AND (Cardiogenic Shock) OR (Cardiac shock) in Title Abstract Keyword AND (((Myocardial Infarct*) OR (Heart Attack)) OR (Acute Coronary Syndrome)) OR (Coronary occlusion) in Title Abstract Keyword - (Word variations have been searched) |
| Embase  13 Sep 2023 | 157 | #4.  #1 AND #2 AND #3  #3.  'extracorporeal membrane oxygenation':ti,ab,kw OR         'extracorporeal life support':ti,ab,kw OR         'extracorporeal circulation':ti,ab,kw OR         'extracorporeal cardiopulmonary         resuscitation':ti,ab,kw  #2.  'cardiogenic shock':ti,ab,kw OR 'cardiac         shock':ti,ab,kw  #1.  'heart infarction':ti,ab,kw OR 'myocardial          infarct':ti,ab,kw OR 'heart attack':ti,ab,kw OR          'acute coronary syndrome':ti,ab,kw OR 'coronary           occlusion':ti,ab,kw |

| Table S2. Details of Quality Assessment Using ROB-II Tools. | | | | | | | |
| --- | --- | --- | --- | --- | --- | --- | --- |
| **Unique ID** | 1 | | **Study ID** | | 1 | **Weight** | 1 |
| **Ref or Label** | Lackermair et al. 2020  (ECLS-Shock-I) | | **Aim** | | assignment to intervention (the 'intention-to-treat' effect) |  |  |
| **Experimental** | ECLS | | **Comparator** | | Standard Care | **Source** | Journal article(s) |
| **Domain** | **Signalling question** | | | | | **Response** | **Comments** |
| **Bias arising from the randomization process** | 1.1 Was the allocation sequence random? | | | | | Y | Yes, the allocation sequence was random using sealed envelopes for randomization. |
|  | 1.2 Was the allocation sequence concealed until participants were enrolled and assigned to interventions? | | | | | Y |  |
|  | 1.3 Did baseline differences between intervention groups suggest a problem with the randomization process? | | | | | N | It is unlikely that the baseline difference in age suggests a problem with the randomization process. |
|  | **Risk of bias judgement** | | | | | **Low** |  |
| **Bias due to deviations from intended interventions** | 2.1.Were participants aware of their assigned intervention during the trial? | | | | | Y | yes carers and people delivering the interventions aware of participants' assigned intervention during the trial  blinidng was not feasible in this case. |
|  | 2.2.Were carers and people delivering the interventions aware of participants' assigned intervention during the trial? | | | | | Y |  |
|  | 2.3. If Y/PY/NI to 2.1 or 2.2: Were there deviations from the intended intervention that arose because of the experimental context? | | | | | N | No deviation from the intended intervention due to the trial context. |
|  | 2.4 If Y/PY to 2.3: Were these deviations likely to have affected the outcome? | | | | | NA |  |
|  | 2.5. If Y/PY/NI to 2.4: Were these deviations from intended intervention balanced between groups? | | | | | NA |  |
|  | 2.6 Was an appropriate analysis used to estimate the effect of assignment to intervention? | | | | | Y | Yes, an appropriate analysis used to estimate the effect of assignment to intervention |
|  | 2.7 If N/PN/NI to 2.6: Was there potential for a substantial impact (on the result) of the failure to analyse participants in the group to which they were randomized? | | | | | NA |  |
|  | **Risk of bias judgement** | | | | | **Low** |  |
| **Bias due to missing outcome data** | 3.1 Were data for this outcome available for all, or nearly all, participants randomized? | | | | | Y | Yes, outcomes' data were available for all randomized participants. All patients completed 12-month follow-up |
|  | 3.2 If N/PN/NI to 3.1: Is there evidence that result was not biased by missing outcome data? | | | | | NA |  |
|  | 3.3 If N/PN to 3.2: Could missingness in the outcome depend on its true value? | | | | | NA |  |
|  | 3.4 If Y/PY/NI to 3.3: Is it likely that missingness in the outcome depended on its true value? | | | | | NA |  |
|  | **Risk of bias judgement** | | | | | **Low** |  |
| **Bias in measurement of the outcome** | 4.1 Was the method of measuring the outcome inappropriate? | | | | | N | Texts do not suggest that the method of measuring the outcome was inappropriate. |
|  | 4.2 Could measurement or ascertainment of the outcome have differed between intervention groups? | | | | | N | Texts do not suggest that measurement or ascertainment of the outcome differed between intervention groups. |
|  | 4.3 Were outcome assessors aware of the intervention received by study participants? | | | | | Y | Texts do not provide information on whether outcome assessors were aware of the intervention received by study participants. However, it is noted that blinding was not feasible in this specific setting due to the nature of the intervention. |
|  | 4.4 If Y/PY/NI to 4.3: Could assessment of the outcome have been influenced by knowledge of intervention received? | | | | | N | The knowledge cannot influence the assessemt of outcome |
|  | 4.5 If Y/PY/NI to 4.4: Is it likely that assessment of the outcome was influenced by knowledge of intervention received? | | | | | NA |  |
|  | **Risk of bias judgement** | | | | | **Low** |  |
| **Bias in selection of the reported result** | 5.1 Were the data that produced this result analysed in accordance with a pre-specified analysis plan that was finalized before unblinded outcome data were available for analysis? | | | | | Y | The study mentioned that the current analysis of prespecified secondary endpoints included 12-month all-cause mortality and the rate of major adverse cardiac event (MACE), suggesting that there was a pre-specified analysis plan in place. |
|  | 5.2 ... multiple eligible outcome measurements (e.g. scales, definitions, time points) within the outcome domain? | | | | | N | No, results are not selected. |
|  | 5.3 ... multiple eligible analyses of the data? | | | | | N | No multiple eligible analyses of the data |
|  | **Risk of bias judgement** | | | | | **Low** | the study mentioned that the current analysis of prespecified secondary endpoints included 12-month all-cause mortality and the rate of major adverse cardiac event (MACE), suggesting that there was a pre-specified analysis plan in place  no results are not selected due multiple eligible outcome measurements (e.g. scales, definitions, time points) within the outcome domain .  no multiple eligible analyses of the data |
| **Overall bias** | **Risk of bias judgement** | | | | | **Low** |  |
| **Unique ID** | 2 | | **Study ID** | | 2 | **Weight** | 1 |
| **Ref or Label** | Banning et al. 2023  (EURO SHOCK) | | **Aim** | | assignment to intervention (the 'intention-to-treat' effect) |  |  |
| **Experimental** | ECLS | | **Comparator** | | Standard Care | **Source** | Journal article(s) |
| **Domain** | **Signalling question** | | | | | **Response** | **Comments** |
| **Bias arising from the randomization process** | 1.1 Was the allocation sequence random? | | | | | Y | Automated web based randomisation done |
|  | 1.2 Was the allocation sequence concealed until participants were enrolled and assigned to interventions? | | | | | Y |  |
|  | 1.3 Did baseline differences between intervention groups suggest a problem with the randomization process? | | | | | N | It does not suggest any baseline differences between the intervention groups that would indicate a problem with the randomization process. |
|  | **Risk of bias judgement** | | | | | **Low** |  |
| **Bias due to deviations from intended interventions** | 2.1.Were participants aware of their assigned intervention during the trial? | | | | | Y | yes carers and people delivering the interventions aware of participants' assigned intervention during the trial  blinidng was not feasible in this case. |
|  | 2.2.Were carers and people delivering the interventions aware of participants' assigned intervention during the trial? | | | | | Y |  |
|  | 2.3. If Y/PY/NI to 2.1 or 2.2: Were there deviations from the intended intervention that arose because of the experimental context? | | | | | N | No deviation from the intended intervention due to the trial context. |
|  | 2.4 If Y/PY to 2.3: Were these deviations likely to have affected the outcome? | | | | | NA |  |
|  | 2.5. If Y/PY/NI to 2.4: Were these deviations from intended intervention balanced between groups? | | | | | NA |  |
|  | 2.6 Was an appropriate analysis used to estimate the effect of assignment to intervention? | | | | | Y | Yes, an appropriate analysis used to estimate the effect of assignment to intervention |
|  | 2.7 If N/PN/NI to 2.6: Was there potential for a substantial impact (on the result) of the failure to analyse participants in the group to which they were randomized? | | | | | NA |  |
|  | **Risk of bias judgement** | | | | | **Low** |  |
| **Bias due to missing outcome data** | 3.1 Were data for this outcome available for all, or nearly all, participants randomized? | | | | | Y | Yes, data for the primary outcome were available for all randomized participants. |
|  | 3.2 If N/PN/NI to 3.1: Is there evidence that result was not biased by missing outcome data? | | | | | NA |  |
|  | 3.3 If N/PN to 3.2: Could missingness in the outcome depend on its true value? | | | | | NA |  |
|  | 3.4 If Y/PY/NI to 3.3: Is it likely that missingness in the outcome depended on its true value? | | | | | NA |  |
|  | **Risk of bias judgement** | | | | | **Low** |  |
| **Bias in measurement of the outcome** | 4.1 Was the method of measuring the outcome inappropriate? | | | | | N | Texts do not suggest that the method of measuring the outcome was inappropriate. |
|  | 4.2 Could measurement or ascertainment of the outcome have differed between intervention groups? | | | | | N | Texts do not suggest that measurement or ascertainment of the outcome differed between intervention groups. |
|  | 4.3 Were outcome assessors aware of the intervention received by study participants? | | | | | Y | Blinding was not feasible in this specific setting due to the nature of the intervention. |
|  | 4.4 If Y/PY/NI to 4.3: Could assessment of the outcome have been influenced by knowledge of intervention received? | | | | | N | The knowledge cannot influence the assessemt of outcome |
|  | 4.5 If Y/PY/NI to 4.4: Is it likely that assessment of the outcome was influenced by knowledge of intervention received? | | | | | NA |  |
|  | **Risk of bias judgement** | | | | | **Low** |  |
| **Bias in selection of the reported result** | 5.1 Were the data that produced this result analysed in accordance with a pre-specified analysis plan that was finalized before unblinded outcome data were available for analysis? | | | | | Y | Pre-specified analysis plan that was finalized before unblinded outcome data were available for analysis. |
|  | 5.2 ... multiple eligible outcome measurements (e.g. scales, definitions, time points) within the outcome domain? | | | | | N | No, results are not selected. |
|  | 5.3 ... multiple eligible analyses of the data? | | | | | N | No multiple eligible analyses of the data |
|  | **Risk of bias judgement** | | | | | **Low** | pre-specified analysis plan that was finalized before unblinded outcome data were available for analysis |
| **Overall bias** | **Risk of bias judgement** | | | | | **Low** |  |
| **Unique ID** | 3 | **Study ID** | | 3 | | **Weight** | 1 |
| **Ref or Label** | Ostadal et al. 2022  (ECMO-CS) | **Aim** | | assignment to intervention (the 'intention-to-treat' effect) | |  |  |
| **Experimental** | ECLS | **Comparator** | | Standard Care | | **Source** | Journal article(s) |
| **Domain** | **Signalling question** | | | | | **Response** | **Comments** |
| **Bias arising from the randomization process** | 1.1 Was the allocation sequence random? | | | | | Y | Automated web based randomisation done |
|  | 1.2 Was the allocation sequence concealed until participants were enrolled and assigned to interventions? | | | | | Y |  |
|  | 1.3 Did baseline differences between intervention groups suggest a problem with the randomization process? | | | | | N | It does not suggest any baseline differences between the intervention groups that would indicate a problem with the randomization process. |
|  | **Risk of bias judgement** | | | | | **Low** |  |
| **Bias due to deviations from intended interventions** | 2.1.Were participants aware of their assigned intervention during the trial? | | | | | Y | yes carers and people delivering the interventions aware of participants' assigned intervention during the trial  blinidng was not feasible in this case. |
|  | 2.2.Were carers and people delivering the interventions aware of participants' assigned intervention during the trial? | | | | | Y |  |
|  | 2.3. If Y/PY/NI to 2.1 or 2.2: Were there deviations from the intended intervention that arose because of the experimental context? | | | | | N | No deviation from the intended intervention due to the trial context. |
|  | 2.4 If Y/PY to 2.3: Were these deviations likely to have affected the outcome? | | | | | NA |  |
|  | 2.5. If Y/PY/NI to 2.4: Were these deviations from intended intervention balanced between groups? | | | | | NA |  |
|  | 2.6 Was an appropriate analysis used to estimate the effect of assignment to intervention? | | | | | Y | Yes, an appropriate analysis used to estimate the effect of assignment to intervention |
|  | 2.7 If N/PN/NI to 2.6: Was there potential for a substantial impact (on the result) of the failure to analyse participants in the group to which they were randomized? | | | | | NA |  |
|  | **Risk of bias judgement** | | | | | **Low** |  |
| **Bias due to missing outcome data** | 3.1 Were data for this outcome available for all, or nearly all, participants randomized? | | | | | Y | Yes, data for the primary outcome were available for all randomized participants. The analysis was performed according to the intention-to-treat principle and included data from all patients for all events that occurred from the time of randomization until 30 days . |
|  | 3.2 If N/PN/NI to 3.1: Is there evidence that result was not biased by missing outcome data? | | | | | NA |  |
|  | 3.3 If N/PN to 3.2: Could missingness in the outcome depend on its true value? | | | | | NA |  |
|  | 3.4 If Y/PY/NI to 3.3: Is it likely that missingness in the outcome depended on its true value? | | | | | NA |  |
|  | **Risk of bias judgement** | | | | | **Low** |  |
| **Bias in measurement of the outcome** | 4.1 Was the method of measuring the outcome inappropriate? | | | | | N | Texts do not suggest that the method of measuring the outcome was inappropriate. |
|  | 4.2 Could measurement or ascertainment of the outcome have differed between intervention groups? | | | | | N | It states that the end points, including the primary end point and safety end points, were reported by investigators without independent adjudication. However, it does not mention any differences in the measurement or ascertainment process between the intervention groups. |
|  | 4.3 Were outcome assessors aware of the intervention received by study participants? | | | | | Y | Blinding was not feasible in this specific setting due to the nature of the intervention. |
|  | 4.4 If Y/PY/NI to 4.3: Could assessment of the outcome have been influenced by knowledge of intervention received? | | | | | N | The knowledge cannot influence the assessemt of outcome |
|  | 4.5 If Y/PY/NI to 4.4: Is it likely that assessment of the outcome was influenced by knowledge of intervention received? | | | | | NA |  |
|  | **Risk of bias judgement** | | | | | **Low** |  |
| **Bias in selection of the reported result** | 5.1 Were the data that produced this result analysed in accordance with a pre-specified analysis plan that was finalized before unblinded outcome data were available for analysis? | | | | | NI | It is not explicitly mentioned whether the data analysis was conducted according to a pre-specified analysis plan that was finalized before unblinded outcome data were available for analysis. |
|  | 5.2 ... multiple eligible outcome measurements (e.g. scales, definitions, time points) within the outcome domain? | | | | | N | No, results are not selected. |
|  | 5.3 ... multiple eligible analyses of the data? | | | | | N | No multiple eligible analyses of the data |
|  | **Risk of bias judgement** | | | | | **Some concerns** | it is not explicitly mentioned whether the data analysis was conducted according to a pre-specified analysis plan that was finalized before unblinded outcome data were available for analysis. |
| **Overall bias** | **Risk of bias judgement** | | | | | **Low** |  |
| **Unique ID** | 4 | **Study ID** | | 4 | | **Weight** | 1 |
| **Ref or Label** | Thiele et al. 2023 | **Aim** | | assignment to intervention (the 'intention-to-treat' effect) | |  |  |
| **Experimental** | ECLS | **Comparator** | | Standard Care | | **Source** | Journal article(s) |
| **Domain** | **Signalling question** | | | | | **Response** | **Comments** |
| **Bias arising from the randomization process** | 1.1 Was the allocation sequence random? | | | | | Y | Automated web based randomisation done |
|  | 1.2 Was the allocation sequence concealed until participants were enrolled and assigned to interventions? | | | | | Y |  |
|  | 1.3 Did baseline differences between intervention groups suggest a problem with the randomization process? | | | | | N | It does not suggest any baseline differences between the intervention groups that would indicate a problem with the randomization process. The document states that the characteristics of the patients were well-balanced between the treatment groups at baseline |
|  | **Risk of bias judgement** | | | | | **Low** |  |
| **Bias due to deviations from intended interventions** | 2.1.Were participants aware of their assigned intervention during the trial? | | | | | Y | yes carers and people delivering the interventions aware of participants' assigned intervention during the trial   blinidng was not feasible in this case. |
|  | 2.2.Were carers and people delivering the interventions aware of participants' assigned intervention during the trial? | | | | | Y |  |
|  | 2.3. If Y/PY/NI to 2.1 or 2.2: Were there deviations from the intended intervention that arose because of the experimental context? | | | | | Y | Yes, there were deviations from the intended intervention that arose because of the trial context. In the trial, crossover to extracorporeal life support (ECLS) therapy in the control group was to be avoided according to the trial protocol. However, in cases of specific predefined criteria for hemodynamic deterioration under medical therapy, escalation therapy using other devices such as an intraaortic balloon pump or a microaxial transvalvular flow pump was allowed. This deviation occurred when there was severe hemodynamic instability with impending hemodynamic collapse, a significant increase in the arterial lactate level, or a substantial increase in vasopressor use to maintain blood pressure . Additionally, a total of 39 patients crossed over from their assigned group to the other group, with 7 of them being in the control group and experiencing refractory cardiac arrest for which ECLS was the only available technique for restoring circulation. |
|  | 2.4 If Y/PY to 2.3: Were these deviations likely to have affected the outcome? | | | | | N | These deviations were not likely to have affected the outcome significantly as the primary analysis was performed according to the intention-to-treat principle, which means that patients were analyzed according to their assigned treatment group regardless of the actual treatment received. Sensitivity analyses were also performed to evaluate the robustness of the data. |
|  | 2.5. If Y/PY/NI to 2.4: Were these deviations from intended intervention balanced between groups? | | | | | NA |  |
|  | 2.6 Was an appropriate analysis used to estimate the effect of assignment to intervention? | | | | | Y | Yes, an appropriate analysis used to estimate the effect of assignment to intervention |
|  | 2.7 If N/PN/NI to 2.6: Was there potential for a substantial impact (on the result) of the failure to analyse participants in the group to which they were randomized? | | | | | NA |  |
|  | **Risk of bias judgement** | | | | | **Some concerns** |  |
| **Bias due to missing outcome data** | 3.1 Were data for this outcome available for all, or nearly all, participants randomized? | | | | | Y | Yes, data for the primary outcome were available for all randomized participants. |
|  | 3.2 If N/PN/NI to 3.1: Is there evidence that result was not biased by missing outcome data? | | | | | NA |  |
|  | 3.3 If N/PN to 3.2: Could missingness in the outcome depend on its true value? | | | | | NA |  |
|  | 3.4 If Y/PY/NI to 3.3: Is it likely that missingness in the outcome depended on its true value? | | | | | NA |  |
|  | **Risk of bias judgement** | | | | | **Low** |  |
| **Bias in measurement of the outcome** | 4.1 Was the method of measuring the outcome inappropriate? | | | | | N | Texts do not suggest that the method of measuring the outcome was inappropriate. |
|  | 4.2 Could measurement or ascertainment of the outcome have differed between intervention groups? | | | | | NI | The document does not specifically provide information on whether the measurement or ascertainment of the outcome differed between intervention groups. However, it does mention that blinding of the intervention was not possible, which may have influenced the therapeutic decisions of treating physicians |
|  | 4.3 Were outcome assessors aware of the intervention received by study participants? | | | | | Y | Blinding was not feasible in this specific setting due to the nature of the intervention. |
|  | 4.4 If Y/PY/NI to 4.3: Could assessment of the outcome have been influenced by knowledge of intervention received? | | | | | PN | However, it does mention that blinding of the intervention was not possible, which may have influenced the therapeutic decisions of treating physicians . Additionally, it states that there were 39 patients who crossed over from their assigned group to the other group, which could have affected the measurement or ascertainment of the outcome |
|  | 4.5 If Y/PY/NI to 4.4: Is it likely that assessment of the outcome was influenced by knowledge of intervention received? | | | | | NA |  |
|  | **Risk of bias judgement** | | | | | **Some concerns** |  |
| **Bias in selection of the reported result** | 5.1 Were the data that produced this result analysed in accordance with a pre-specified analysis plan that was finalized before unblinded outcome data were available for analysis? | | | | | Y | Yes, the data that produced this result were analyzed in accordance with a pre-specified analysis plan that was finalized before unblinded outcome data were available for analysis. The document states that the trial protocol was approved by the ethics committee and designed by the first author, and it was further modified by the steering committee. |
|  | 5.2 ... multiple eligible outcome measurements (e.g. scales, definitions, time points) within the outcome domain? | | | | | N | No, results are not selected. |
|  | 5.3 ... multiple eligible analyses of the data? | | | | | N | No multiple eligible analyses of the data |
|  | **Risk of bias judgement** | | | | | **Low** | Yes, the data that produced this result were analyzed in accordance with a pre-specified analysis plan that was finalized before unblinded outcome data were available for analysis. The document states that the trial protocol was approved by the ethics committee and designed by the first author, and it was further modified by the steering committee. |
| **Overall bias** | **Risk of bias judgement** | | | | | **Some concerns** |  |
